# Digital twin system for manufacturing processes based on a multi-layer knowledge graph model

**DOI:** 10.1038/s41598-024-85053-0

**Published:** 2025-04-14

**Authors:** Chang Su, Xin Tang, Qi Jiang, Yong Han, Tao Wang, Dongsheng Jiang

**Affiliations:** 1 Department of Information Science and Engineering, Ocean University of China, Qingdao, 266100 China; 2 Laboratory for Regional Oceanography and Numercial Modeling, Qingdao Marine Science and Technology Center, Qingdao, China; 3 Control and Computer Engineering, North China Electric Power University, Beijing, 102206 China; 4 Institute of Computing Technology, Chinese Academy of Sciences, Beijing, 100190 China; 5 AECC South Industry Company Limited, Lusong District, Zhuzhou City, 410000 Hunan Province China

**Keywords:** Digital twin, Knowledge graph, Intelligent manufacturing, Data integration, Manufacturing process management, Decision support, Mechanical engineering, Electrical and electronic engineering

## Abstract

Digital twin technology in the manufacturing process faces challenges like integrating diverse data sources and managing real-time data flow. To address this, we propose a novel three-layer knowledge graph architecture to enhance digital twin modeling for manufacturing processes. This architecture consists of a concept layer that structures key information into a knowledge network, a model layer that aligns digital and physical parameters, and a decision layer that leverages model and real-time data for decision support. Validated in aero-engine blade production, this system integrates multi-source data, enhances predictive analysis and anomaly detection, and supports process control and quality management. Over a 5-month validation period, the maximum contour error precision of the blades improved from 0.073 mm to 0.062 mm, and the product qualification rate increased from 81.3% to 85.2%. This demonstrates the system’s robust capability for advancing digital twin utilization in manufacturing, highlighting its potential for future improvements.

## Introduction

Against the backdrop of Industry 4.0 and smart manufacturing, enterprises are grappling with challenges such as increasing production efficiency, reducing manufacturing costs, optimizing processes, and implementing real-time monitoring. Digital twin technology, an innovative fusion of virtual and real-world elements, has gained significant attention in recent years. As virtual representations of physical devices, systems, or processes, digital twins enable seamless integration of data with physical entities, effectively supporting the optimization and monitoring of production processes^1^. The potential of digital twins in industrial applications extends beyond just enhancing efficiency and reducing costs; they also improve product quality, support decision-making, and enable real-time monitoring of equipment and production lines. By predicting potential faults and formulating proactive measures, digital twin technology ensures the stability and reliability of production processes^2,3^. However, practical deployment presents multiple challenges. Collecting and integrating heterogeneous data from diverse sensors, controllers, and devices makes constructing an accurate digital model exceedingly complex. Additionally, the difficulty in interacting with contextual information poses significant hurdles in real-time data transmission and instantaneous model feedback. Digital twin models must not only precisely map real-world devices, systems, and processes but also continuously adjust and update in response to changes in production conditions, equipment wear, and manufacturing process updates to remain synchronized with the real world. Thus, finding a balance between ensuring real-time information and maintaining model accuracy to meet the stringent demands of industrial applications becomes an urgent issue to address^4^.

## Related work

### Industrial digital twin

Digital twin technology, which fully utilizes physical models, sensor updates, operational history, and other data, integrates multidisciplinary, multi-physical, multiscale, and multiprobability simulation processes to map virtually the entire lifecycle of corresponding physical entities. Thus, the digital twin technological system must support elements such as virtual and physical spaces, as well as bidirectional information flow, playing a crucial role throughout the entity’s lifecycle^5^. The utilization of digital twin technology in industrial applications is rapidly growing, particularly showing significant potential in smart manufacturing and Industry 4.0. Tao et al.^6^ provided a comprehensive overview of digital twins, emphasizing their capability in real-time monitoring and optimization of manufacturing systems. Additionally, Qi and Tao^7^ explored the integration of digital twins with modern manufacturing techniques to enhance production efficiency and system reliability. The real-time data synchronization and precise modeling capabilities of digital twins are crucial for optimizing production lines. Lu et al.^8^ demonstrated the application of digital twins in complex industrial settings, showing how dynamic data analysis can improve operational and maintenance strategies. Lohtander et al.^9^ discussed using Micro Manufacturing Units (MMU) as a research environment for digital twins, aiming to deepen the understanding of digital twin modeling and information requirements. Qi et al.^10^ introduced a five-dimensional digital twin model as a reference guide for implementing digital twins, focusing on enabling technologies and tools. Leng et al.^11^ displayed a digital twin-driven cyber-physical system for managing smart workshops, showcasing its application in a smart manufacturing environment. Wang et al.^12^ proposed a digital twin reference model for fault diagnosis in rotating machinery, highlighting the importance of parameter sensitivity analysis for enhancing model adaptability. Tong et al.^13^ presented a real-time machining data application and service based on an IMT digital twin, demonstrating the effectiveness of the development method. Melesse et al.^14^ conducted a systematic literature review on Digital Twin models in industrial operations, emphasizing the role of Digital Twins in coupling physical systems with virtual representations. Yu et al.^15^ proposed a Digital Twin approach for health monitoring using a nonparametric Bayesian network to model the dynamic degradation process and uncertainty propagation.

In summary, although digital twin technology offers numerous advantages and the industrial digital twin model technology has rapidly evolved, challenges remain when deploying on a large scale in industrial production, including the complexity of heterogeneous data integration and multi-source information management issues.

### Knowledge graph in digital twin

Knowledge graph^16^, as a structured method of representing knowledge, describe the real-world information through entities, their attributes, and relationships. They support semantic querying and reasoning, thereby enhancing data discovery, connectivity, and utilization. The core of knowledge graphs lies in their ability to integrate and connect a vast array of heterogeneous data sources, providing a rich semantic context for machine learning models and decision support systems. Zhao et al.^17^ proposed a collaborative approach for developing open industrial knowledge graphs to gather knowledge across domains, incorporating tiered matching procedures. Furthermore, Zhao et al.^18^ emphasized the importance of knowledge graphs in driving the next industrial technology revolution, particularly in the automotive sector, by extracting triples automatically from unstructured Chinese text using traditional NLP and deep learning methods. Yakovlev et al.^19^ highlighted the necessity of creating a classification with geomechanically specific phenomena based on the construction of an industrial knowledge graph in the context of coal field development. Moreover, Zhou et al.^20^ introduced a knowledge representation model that associates entities and relations to form an industrial knowledge graph, enhancing knowledge retrieval and question-answering methods. Recent studies have focused on leveraging industrial knowledge graphs to promote the development of cognitive manufacturing networks. Zheng et al.^21^ proposed a multi-agent reinforcement learning approach based on an industrial knowledge graph, while Li et al.^22^ discussed achieving cognitive mass personalization through a self-X cognitive manufacturing network enabled by industrial knowledge graphs and graph embeddings. Additionally, Weihua et al.^23^ conducted a bibliometric analysis of industrial knowledge graph research, revealing that the field is still evolving with hotspots in knowledge graphs, knowledge representation, and the application of knowledge graphs in modeling and industrial management innovation. Lyu et al.^24^ introduced a crowdsourcing-based continuous enrichment method for industrial knowledge graphs to achieve Knowledge-as-a-Service in IIoT-driven smart manufacturing. Industrial knowledge graphs play a crucial role in enhancing information support, knowledge retrieval, and decision-making processes^25^. Knowledge graphs are increasingly applied in the development of digital twin models across various sectors. Banerjee et al.^26^ proposed a method using a graph-based query language enriched with inference rules to formalize knowledge as digital twin models for industrial production lines. Jiang et al.^27^ proposed a digital twin system structure for assembly processes based on a knowledge graph to record actual process data. Akroyd et al.^28^ discussed a dynamic knowledge graph approach for digital twins, particularly focusing on developing a digital twin of the UK to support energy system decarbonization. Sahlab et al.^29^ suggested a Knowledge Graph-enhanced architecture for intelligent Digital Twins, offering capabilities such as internal linking, knowledge completion, error detection, collective reasoning, and semantic querying. Su et al.^30^ introduced a knowledge-based digital twin system that leverages a comprehensive knowledge graph to construct a digital twin model for the manufacturing process. This approach enables precise description, management, prediction, and optimization of the process through knowledge matching and inference within the knowledge, geometry, and decision models.

In summary, as the maturity of knowledge graphs in industrial applications gradually increases, more scholars are beginning to explore the application of knowledge graph technology in the field of industrial digital twins, leveraging its powerful information management capabilities. Although current research is still in its initial stages, it has shown tremendous potential for development and application prospects, with many technical challenges and issues expected to be resolved in the future.

## Methodology

**Fig.1:**
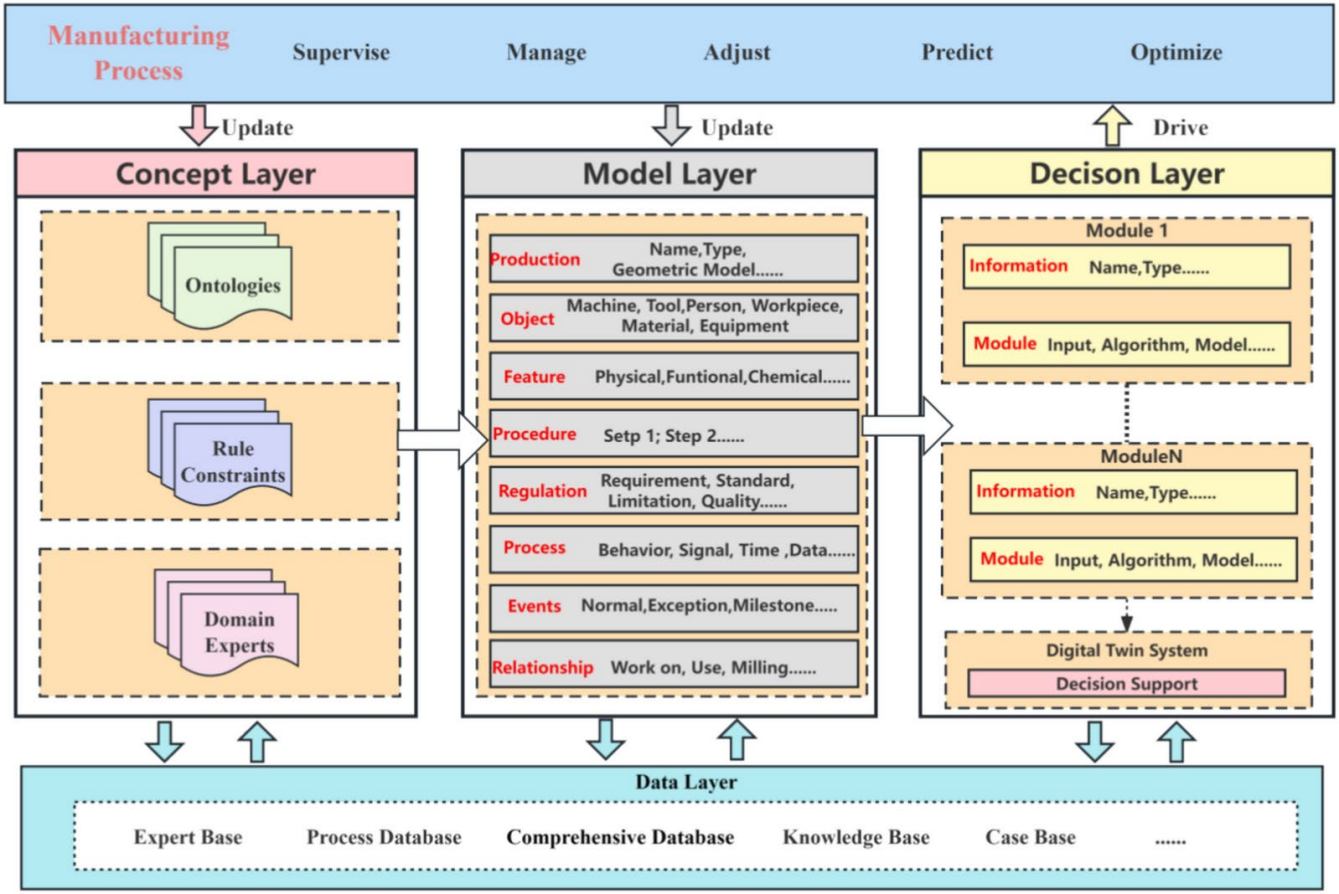
Whole architecture of multi-layer knowledge graph.

This section introduces a three-layer digital twin knowledge graph model consisting of the concept layer, model layer, and decision layer, as illustrated in Fig. 1. Starting from the entire industrial processing cycle, the model undertakes a thorough modeling of physical elements and algorithm applications, deeply analyzing three levels: conceptual abstraction, data instantiation, and decision support implementation. This multi-layered, comprehensive, and dynamic model architecture allows for more precise and detailed modeling and analysis of industrial processing knowledge.

In the concept layer, a universal knowledge graph for the manufacturing domain is constructed by integrating an ontology repository, rule constraints, and domain expert knowledge. This layer provides all the foundational knowledge and frameworks necessary for the model, ensuring the standardization and logical consistency of the knowledge. The model layer instantiates the knowledge from the concept layer, acquiring data and performing digital modeling for specific product objects. The knowledge graph in this layer is transformed into an applicable product knowledge graph, and the constructed digital information model serves as the virtual model of the product, used to simulate and analyze the actual working state and performance of the product. Finally, the decision layer establishes a knowledge graph related to various decision-making functions of the twin system, including the inputs from the model layer’s digital twin models and real-time manufacturing data as inputs to implement decision support within different application modules. The key in this layer lies in driving real-time information interaction between the virtual model and the physical system, ensuring timely data updates and decision support, thereby enhancing the system’s response speed and processing capabilities. As the manufacturing process continues, the ongoing data interaction between the model and the system updates the knowledge and data, which is fed back into the model, continually updating and optimizing the current model. Through this multi-layered architecture, the model not only accurately maps and optimizes the actual industrial processing operations but also flexibly adapts to the evolving production demands and conditions.

### Concept layer

**Fig.2:**
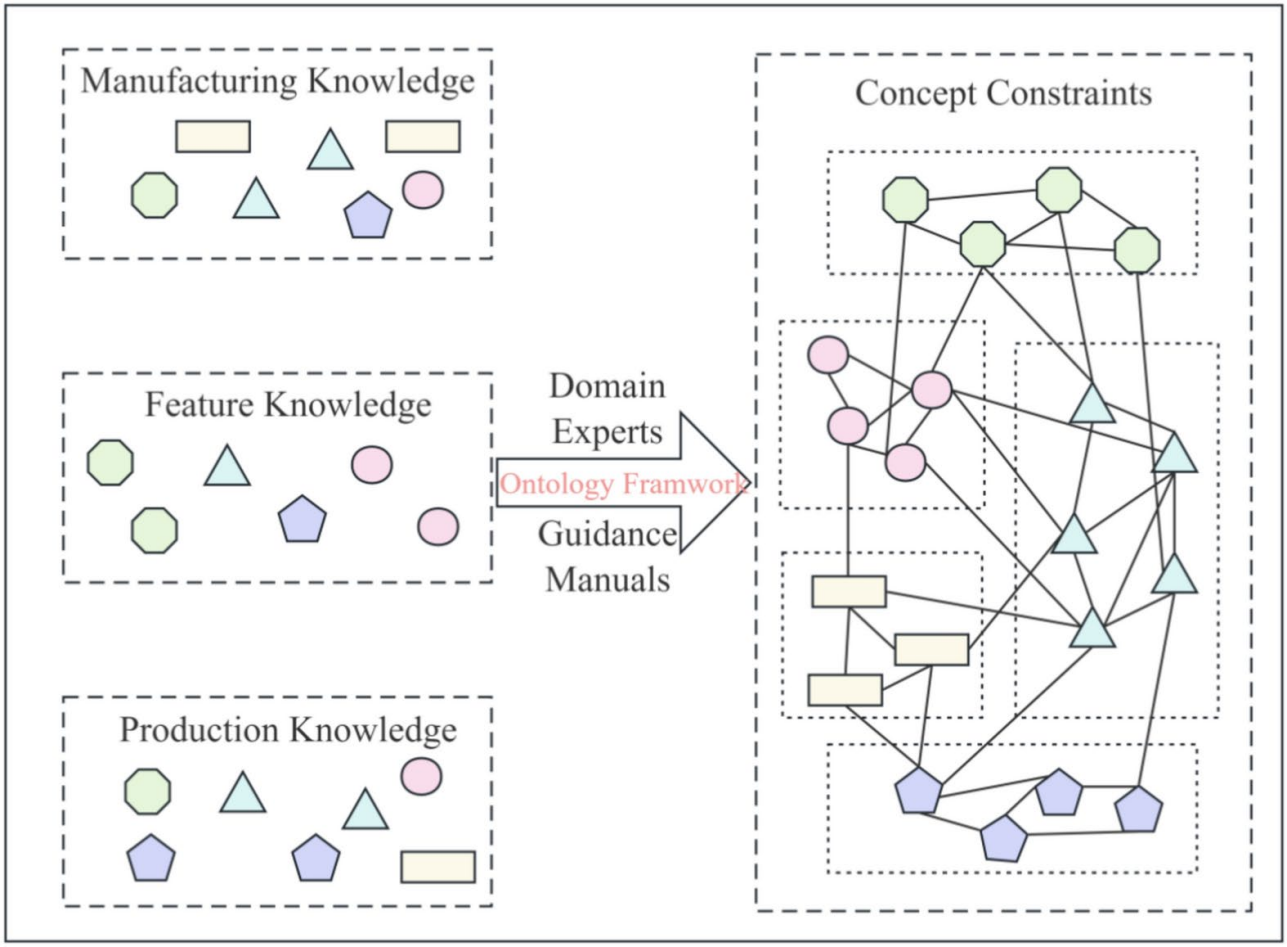
Concept layer architecture.

The concept layer serves as the foundational tier of the multi-level knowledge graph, focusing on building an ontology library tailored for the industrial manufacturing domain. As depicted in Fig. 2, this layer adopts ontological frameworks, providing a comprehensive theoretical foundation through the structured categorization of knowledge. The construction of the ontology library draws on the deep insights of domain experts and incorporates practical knowledge from various technical manuals and guidelines, ensuring the richness and accuracy of the domain’s ontological concepts. Through such methodology, the concept layer successfully constructs a universal knowledge framework representative of the industrial manufacturing field. This framework extensively covers the fundamental concepts relevant to the domain, laying a solid foundation for the further development and implementation of the digital twin model. Additionally, it offers a method to support the integration of complex data and rules. The architecture of the concept layer can be detailed as follows:

1 $$Concept = \{ C_{id} ,R_{T} ,R_{A} ,C_{R} ,C_{I} ,C_{C} \}$$

$C_{id}$ represents the unique identifiers of basic concepts, ensuring that each concept within the model can be accurately identified and distinguished, supporting the structured management and querying of the model. These concepts form the core of the industrial manufacturing domain’s knowledge graph, providing the foundational framework and parameters for the digital twin model. $R_{T}$ defines the types of rules, which dictate the interactions and constraints between concepts. It illustrates the diversity and complexity of the rules within the concept layer, encompassing but not limited to operational rules, safety standards, and quality control measures within the manufacturing process. $R_{A}$ denotes the scope of application for each rule. It specifies the domain and impact of each rule within particular application scenarios, such as rules applicable to specific product lines, manufacturing stages, or operational environments. $C_{R}$ represents the collection of basic elements defined by specific rules. It reveals the logical relationships and structural connections between these elements, forming an interconnected network within the knowledge graph that provides a logical basis for data interaction and functional implementation of the digital twin model.$C_{I}$ describes the dependencies between concepts, which are crucial for understanding the coordination and optimization of various steps within the production process.$C_{C}$ describes the correlations between concepts, used to assess the degree of association and interactive impact of different concepts within specific industrial applications. By analyzing and determining the inter-concept correlations, the connections within the knowledge graph can be more effectively constructed, optimizing information flow and decision-making processes. This element is particularly important in revealing key concepts that interact or depend on each other within production lines or manufacturing processes, thus enhancing the predictive and analytical capabilities of the digital twin model.

### Model layer

**Fig.3:**
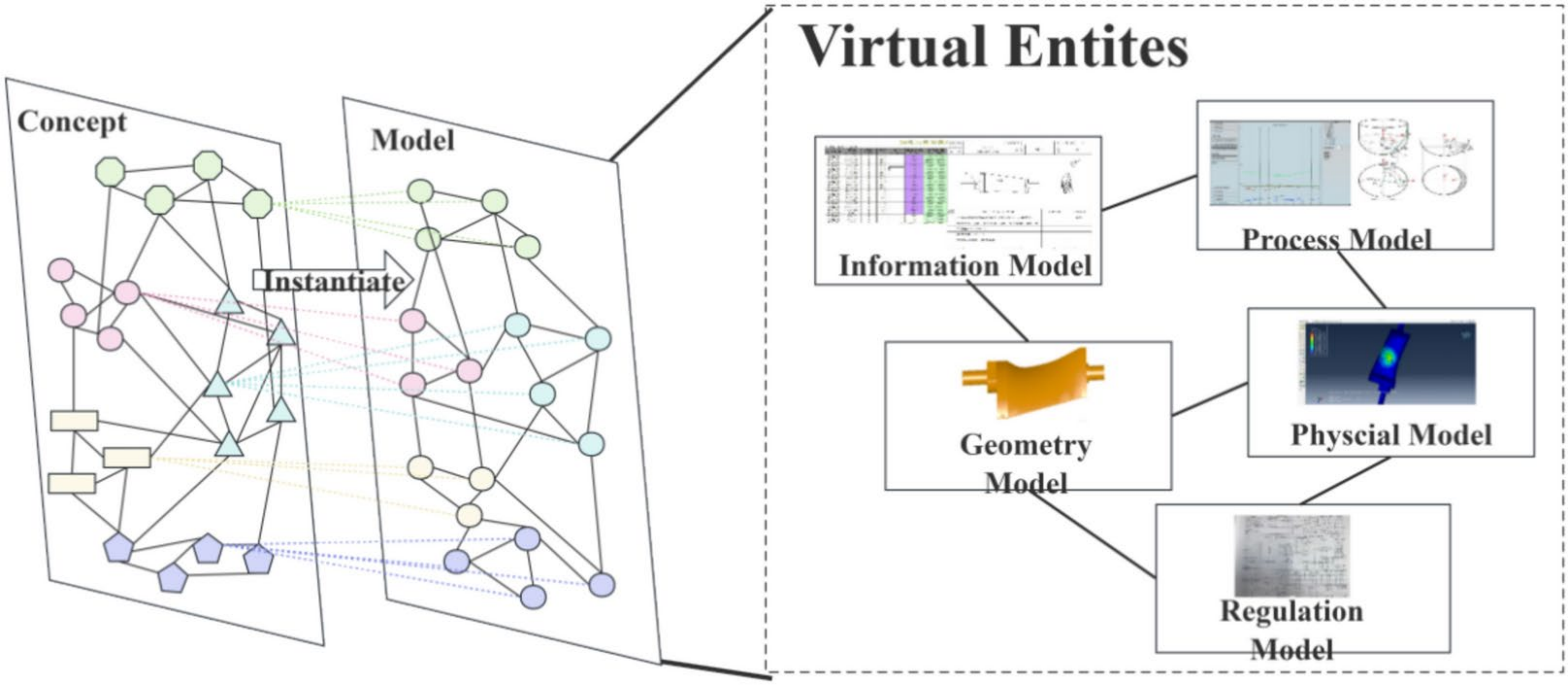
Model layer architecture.

The model layer focuses on specific product models within the industrial processing domain, instantiating the rules and constraints defined in the concept layer and performing detailed digital modeling of their physical characteristics during the manufacturing process, as illustrated in Fig. 3. This layer provides crucial data support for the physical entities of the twin model, integrating physical data and theoretical parameters from the actual product manufacturing process into the conceptual model to enable more accurate simulation and analysis of real situations. In constructing the model layer, strict adherence is maintained to the interactive relationships and constraints among elements identified in the concept layer, through comprehensive analysis of each element’s physical properties, states, specifications, and common attributes, ensuring the model’s completeness and accuracy, and providing a precise and reliable digital representation for the product manufacturing process. The representation of the model layer can be summarized as follows:

2 $$Model = \{ M_{id} ,M_{T} ,M_{C} ,M_{U} \}$$

$M_{id}$ represents the unique identifier for each instantiated product within the model, ensuring the independence and identifiability of each product instance within the model. $M_{T}$ denotes the type of model entity, corresponding to the type of elements in the concept layer, maintaining consistency and connection between the entity and its original concept. $M_{C}$ describes the common attribute set of model entities, which are the characteristic attributes shared by similar elements during the modeling process, used to demonstrate the similarity among model entities. For example, all product models share static attributes determined before manufacturing, including standardized manufacturing steps, machine tools, and tools used. $M_{U}$ represents the unique attribute set of model entities, which are specific characteristic attributes each model entity possesses during instantiation. In the actual manufacturing process, each product instance generates dynamic data based on specific operational conditions and environment. These attributes reflect the specific conditions and personalized parameters of each product instance during processing, highlighting the model’s uniqueness and functional features in specific application environments.

### Decision layer

**Fig.4:**
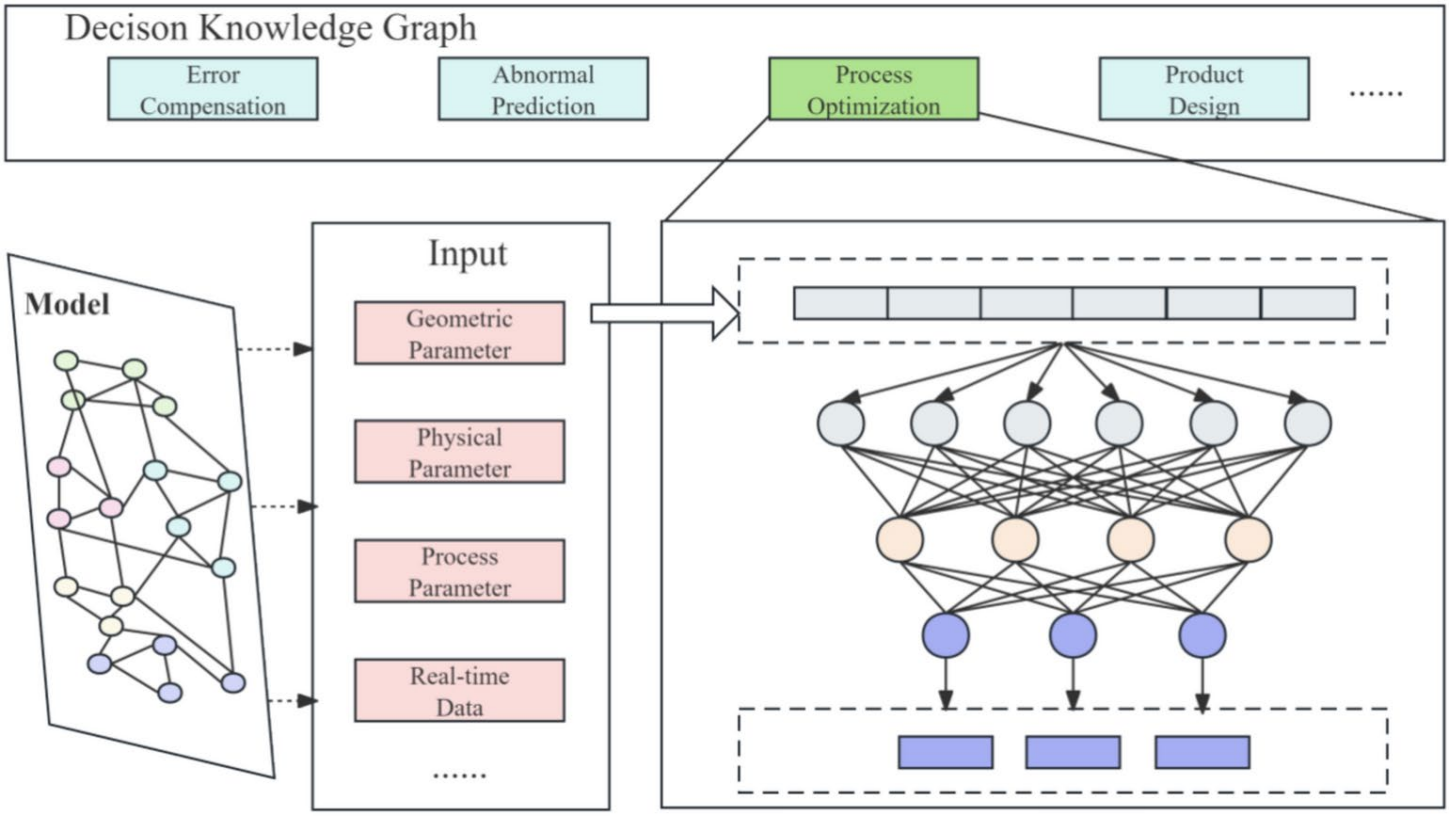
Decision layer architecture.

The decision layer represents the practical application phase of the digital twin model within the physical world, leveraging the detailed data provided by the model layer to execute various decision-making tasks. As shown in Fig. 4, this layer primarily focuses on implementing dynamic optimization, maintenance forecasting, and risk management to ensure the smooth and efficient operation of the manufacturing process. Utilizing the precise digital model constructed by the model layer, the decision layer conducts in-depth analyses and utilizes this data and knowledge for complex computations and predictive analytics, aiming to identify and resolve potential operational issues promptly to optimize overall manufacturing operations. The knowledge graph architecture of this layer not only enhances the decision-making capabilities of the twin model but also, through advanced data analysis and machine learning technologies, enables the digital twin system to respond quickly and accurately to various variables in the industrial environment, improving the manufacturing system’s adaptability and decision-making quality. The architecture and operation of the decision layer are described in detail as follows:

3 $$Decision = \{ D_{id} ,D_{T} ,D_{M} \}$$

$D_{id}$ represents the unique identifier for decision, used to identify and distinguish between different application modules. $D_{T}$ indicates the type of decision, displaying the role of the function module within the digital twin system and its targeted specific decision support. $D_{M}$ represents the detailed in function modules used for decision, which integrate the latest algorithms and technological methods to effectively process data, execute predictions, and optimize manufacturing process. The computation of $D_{M}$ can be described as follows:

4 $$D_{M} = \{ M_{S} ,M_{I} ,M_{O} \}$$

$M_{S}$ represents the algorithmic structure of the module, detailing the types and configurations of algorithms, including neural network architectures and optimal hyperparameters. $M_{I}$ denotes the functional inputs of the module, including the required data types and pre-processing conditions. $M_{O}$ represents the output of the module, serving as the interface between the digital twin system and the model, with the output results used to guide subsequent decision-making and operations in the twin system.

In the decision-making process of production, a substantial amount of real-time data is generated from various sensors and equipment on the manufacturing floor. Processing this high-frequency data efficiently presents a significant challenge, especially when dealing with large-scale data updates that occur in milliseconds. To address this issue, the digital twin system incorporates a scalable and efficient real-time data processing architecture that ensures timely and accurate reflections of the manufacturing process within the digital model. The system employs an integrated approach that combines edge computing and cloud computing resources to manage real-time data effectively. At the edge level, data processing is performed close to the data sources, which includes initial tasks such as data filtering, noise reduction, and preliminary anomaly detection. By handling these processes locally on edge devices, the system reduces latency and minimizes the volume of data that needs to be transmitted over the network. This local processing is crucial for managing high-frequency updates, as it allows for immediate responses to critical events on the production line without overwhelming the network infrastructure. After the initial processing at the edge, the refined data is transmitted to a cloud platform for more intensive computations and advanced analytics. The cloud infrastructure leverages distributed data processing frameworks capable of handling large-scale data streams. Apache Kafka is utilized for data streaming, enabling the system to handle continuous flows of data efficiently. For real-time analytics, frameworks like Apache Spark is employed, which allow for scalable and fault-tolerant processing of vast amounts of data in real time. To maintain low latency and high throughput, the architecture employs several strategies. In-memory data processing techniques are used to accelerate data analysis and model updates, reducing the time required to access and compute data. Parallel computing is implemented to distribute workloads across multiple processing units, allowing the system to handle vast amounts of data simultaneously. This parallelism ensures that the digital twin models are updated promptly, reflecting the current state of the manufacturing process accurately. The system also supports horizontal scaling, which means that additional computational resources can be added dynamically in response to increasing data loads. This scalability ensures consistent performance even as the volume of real-time data grows. Load balancing mechanisms are in place to distribute incoming data evenly across processing nodes, preventing bottlenecks and ensuring that no single node becomes a point of failure. By integrating edge computing for immediate, localized data processing with cloud computing for more complex analytics, the digital twin system effectively manages high-frequency data updates and large-scale data processing demands. This comprehensive approach ensures that the digital twin remains synchronized with the physical manufacturing environment, enabling real-time monitoring, predictive analytics, and timely decision-making. Consequently, the system enhances manufacturing efficiency, reduces downtime, and improves product quality by providing accurate and up-to-date information for process optimization.

**Fig.5:**
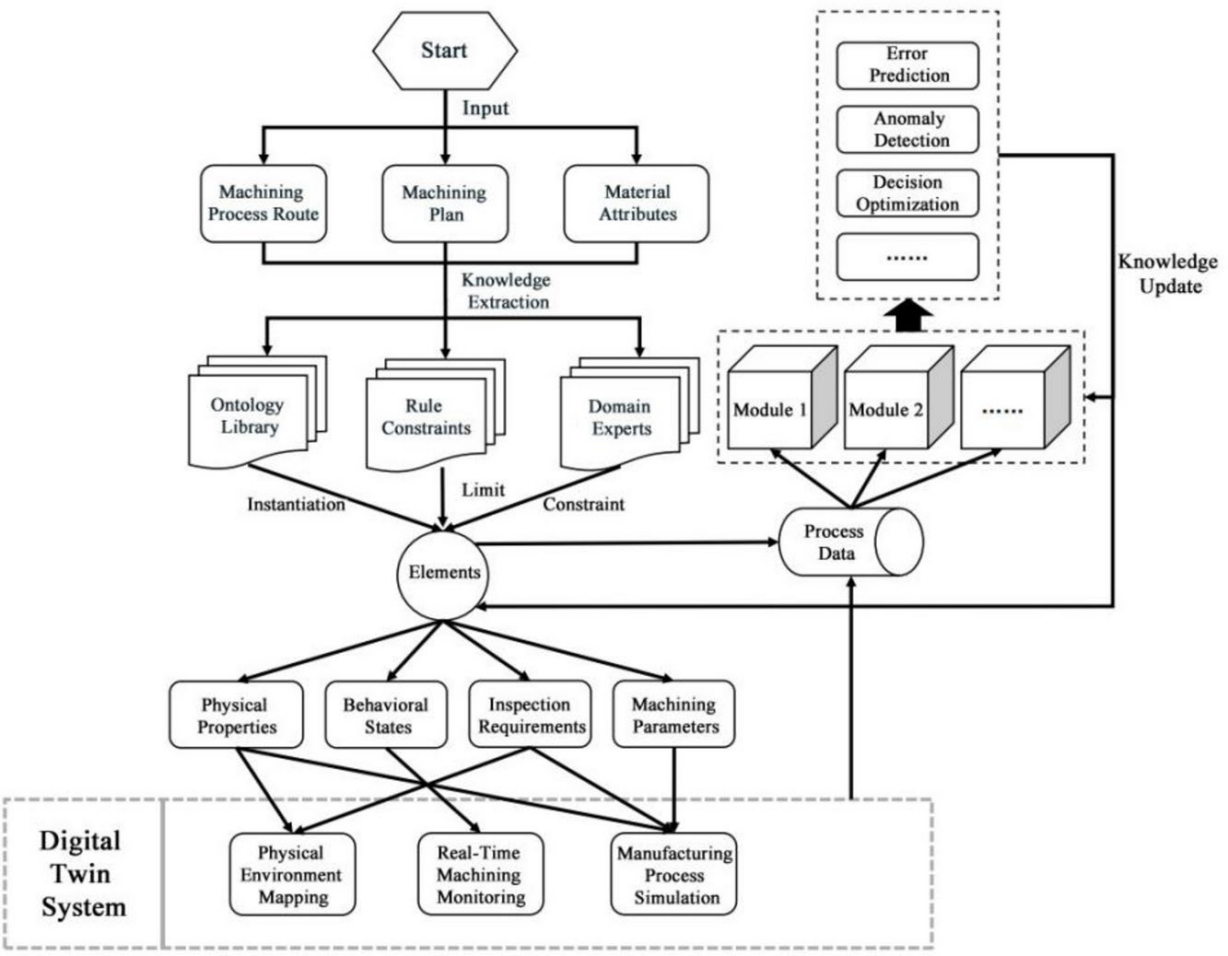
Self-update and iteration of digital twin model.

Through the decision layer, the digital twin knowledge graph is not merely a static repository of data but transforms into a dynamic, intelligent system capable of real-time response and optimization in the manufacturing process. This architecture ensures that every phase, from concept to model and onto practical application, is supported and enhanced, thus elevating efficiency and reducing operational risks in industrial manufacturing. The application of this three-layer architecture not only augments the technological capabilities of enterprises but also lays a robust foundation for achieving more advanced operational management and decision support. As illustrated in Fig. 5, data for the digital twin knowledge graph of the manufacturing process generally originates from machining process routes, machining plans, and material attributes. For unstructured and semi-structured data, this approach utilizes knowledge extraction to derive ontology libraries, machining rules, and behavioral constraints, integrating input from domain experts. Using the ontology library, elements are instantiated and regulated based on machining rules and behavioral constraints. The digital twin system uses the physical properties and material specifications of the instantiated elements to map the physical environment of the digital twin; monitors real-time machining status based on the behavioral states of elements; and simulates the industrial machining process by leveraging physical environments combined with relevant parameters of the element’s machining process. The process data generated by the digital twin system’s simulation of the machining process and real-time machining status monitoring serve as inputs to various decision-making modules. The decision layer not only carries out predictive and optimization tasks but also drives system functions through these processes to achieve dynamic optimization of the manufacturing process. By using the precise simulation data provided by the model layer, the decision layer can monitor the production process in real-time, predict potential faults or efficiency bottlenecks, and make necessary adjustments to optimize the operation of the production line. More importantly, this process is continuous and cyclical. With ongoing data input and the integration of real-time feedback, the digital twin model continually updates and adjusts itself based on actual operating conditions and external changes. This dynamic update of the model is not limited to reacting to the current production conditions but also includes predicting future changes through algorithmic models, thereby making the manufacturing process more intelligent and adaptable. Furthermore, this continual model optimization and data updating mechanism ensures that the digital twin system evolves over time, adapting to new manufacturing technologies and market demands. This iterative process, through continuous learning and adaptation, enhances the accuracy of decisions and the efficiency of manufacturing processes.

## Case study

**Fig. 6:**
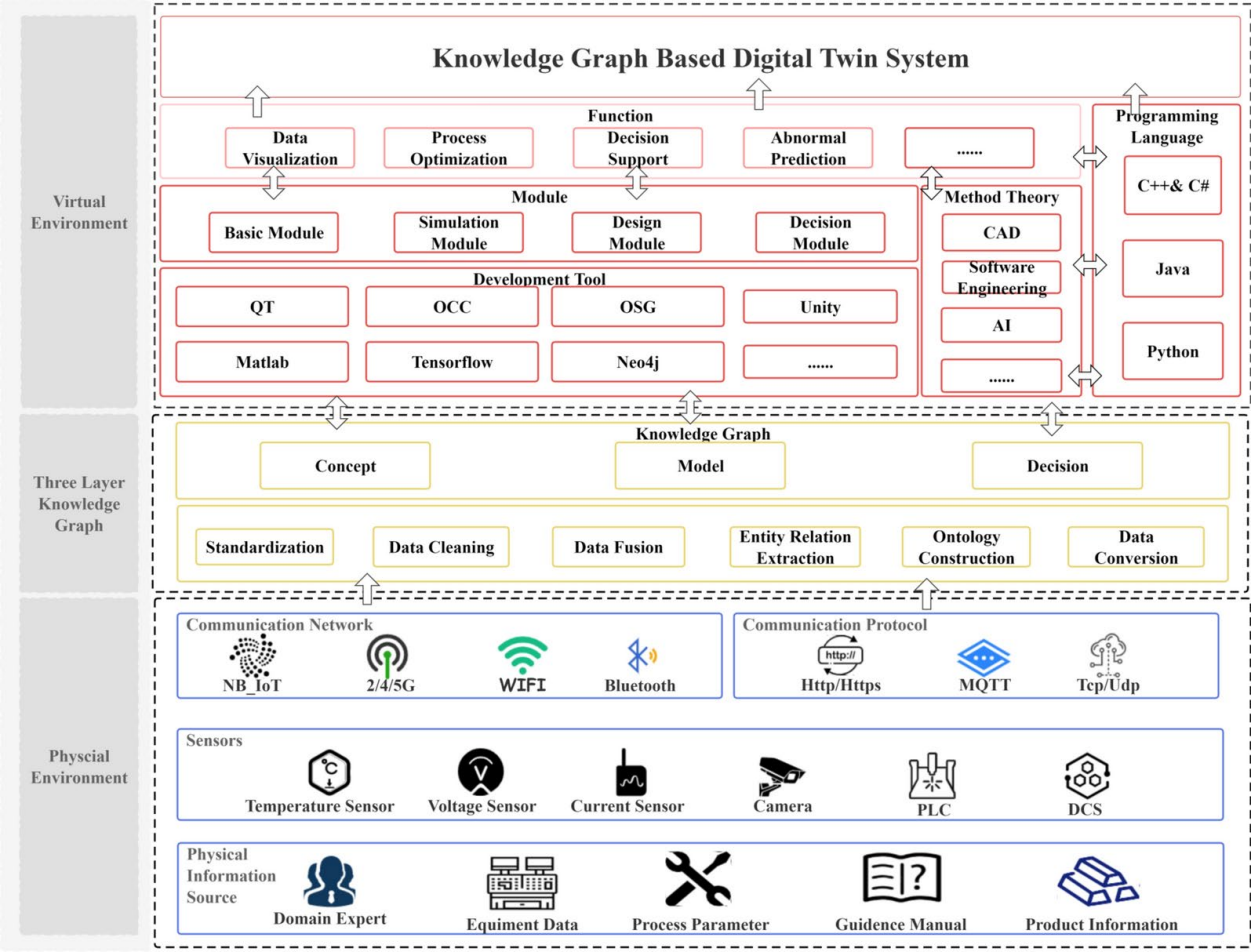
Manufacturing process digital twin architecture.

Based on the three-layer knowledge graph architecture proposed in this study, a digital twin architecture for the manufacturing process is presented as shown in Fig. 6. This architecture is divided into three parts: the physical environment, the three-layer knowledge graph, and the virtual environment. The physical environment refers to the actual manufacturing process, where various sensors deployed in machines and other equipment collect data from the manufacturing operations. Additionally, domain experts and related process manuals provide rules and constraints to construct a conceptual framework for the manufacturing process. In this physical setting, diverse and heterogeneous data and information are transmitted to the three-layer knowledge graph through various IoT technologies, constructing the conceptual framework, digital model, and decision-support model of the manufacturing process. The three-layer knowledge graph establishes an interface for information exchange between the physical and virtual environments, allowing for precise interaction. In the virtual environment, a semantic digital twin software tool is developed based on the information from the three-layer knowledge graph, utilizing various computer theories and software engineering technologies and tools. This tool facilitates the construction and simulation of virtual models and the optimization and prediction of relevant data. Through the formulation of relevant decisions, optimized and updated knowledge is fed back into the knowledge graph, thereby enabling the iterative optimization of the model and influencing the actual manufacturing process.The relevant case studies will be conducted within the system based on this architecture.

Domain Ontology Library refers to the formalized expression of concepts and the relationships between them within a specific domain, essentially serving as a shared resource description mechanism. It is used to describe the knowledge information within a domain, typically described using the OWL (Web Ontology Language). OWL2RL allows for the declaration of equivalent classes and local constraints on properties, extending the expressive capabilities of RDFS. This enables a better representation of the complex relationships involved in the manufacturing processes of intricate product components. Therefore, this paper opts to use the OWL2RL language to establish a process ontology library for product manufacturing. Table 1 lists the main vocabularies of OWL RL and provides application examples.

**Table1:** OWL RL vocabularies.

The main vocabulary	Example
rdfs:subClassOf	Milling machine ⊆ Machine tool
rdfs:sameClassAs	NC ≡ Turning ∪ Grinding ∪ Milling
rdfs:subPropertyOf	Using a ball-end mill ⊆ Using a tool
rdfs:domain	∃Manufactured by ,T ⊆ Blade
rdfs:range	T ⊆ ∀Manufactured by , Blade Material
owl:disjointWith	Turning tool ∩ Broaching tool ⊆ ⊥
owl:inverseOf	Causing change ≡ Occurring change
owl:symmetricProperty	Similarity
owl:hasValue	Ball-end milling cutter ≡ Tool model value ‘001’

**Fig. 7:**
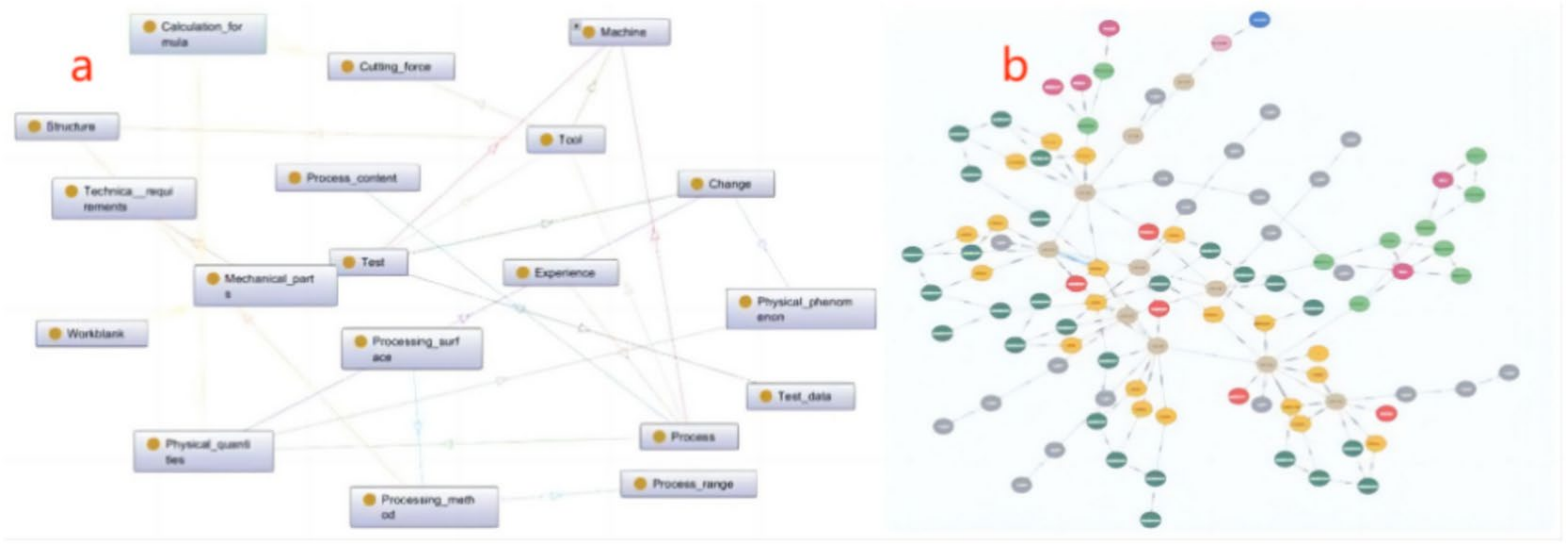
(**a**) Manufacturing process ontology(partial); (**b**) Aero-engine blade manufacturing process knowledge graph (partial).

As illustrated in Fig. 7(a), a relevant conceptual ontology for the manufacturing process was developed under the guidance of domain experts and procedural manuals. (b) depicts the knowledge graph of the manufacturing process for the aero-engine blade, which is built on the foundation of the conceptual ontology. This process led to the creation of a digital information model for the blade manufacturing process.

**Fig. 8:**
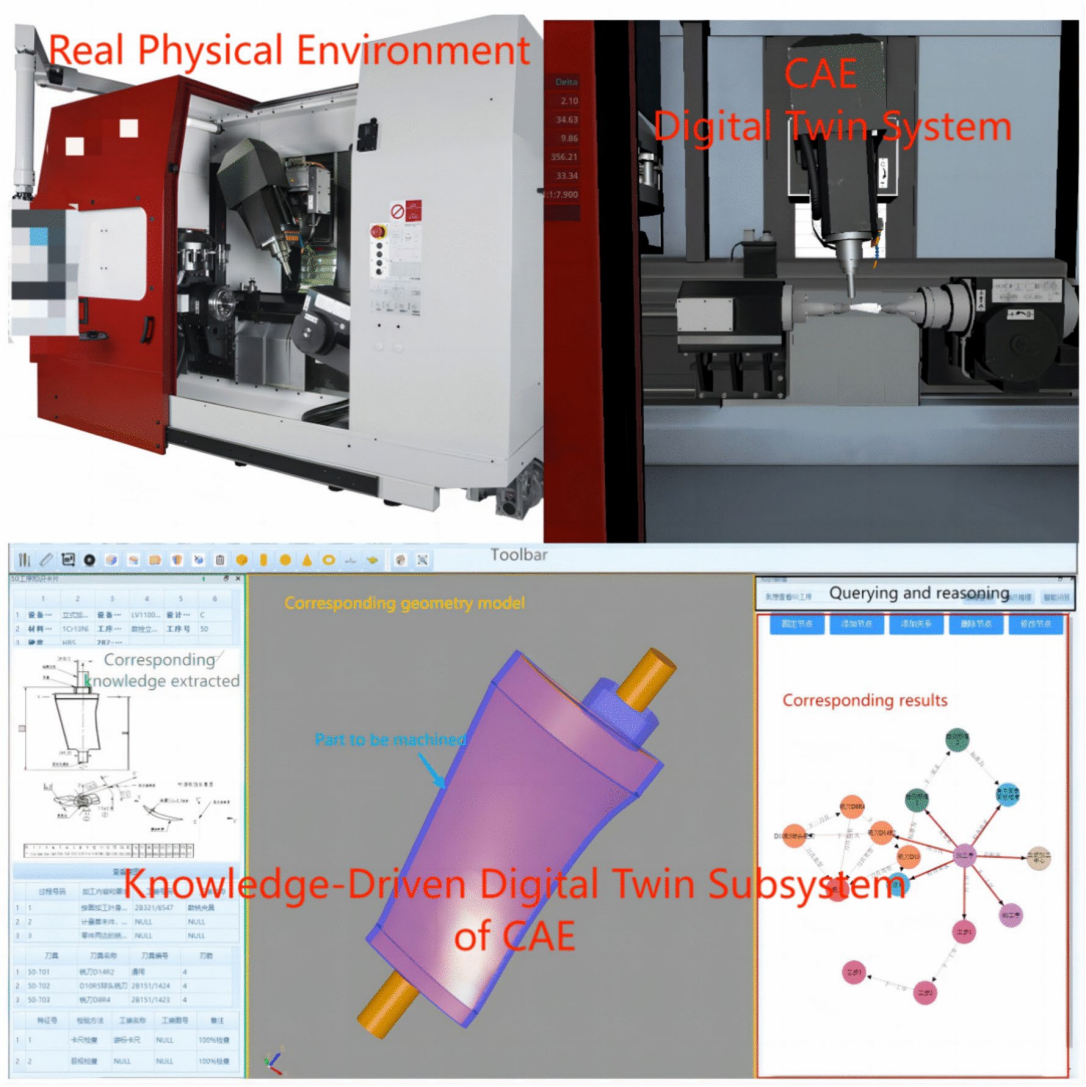
Digital twin system and physical environment in manufacturing process.

Illustrated in Fig. 8, the real physical environment and the CAE digital twin system. In the real physical environment, we observe a complex machine tool apparatus capable of precision machining. Correspondingly, the CAE digital twin system demonstrates how advanced simulation and data analysis can accurately reproduce the operations and performance of the physical machine tool in a digital space, aiding in the optimization of manufacturing processes and the early prediction of potential issues. Additionally, this CAE system includes a knowledge-driven digital twin subsystem for product manufacturing processes, built around an advanced knowledge graph. This subsystem focuses on the twin model of the products machined by the tool, enabling real-time synchronization of machine-related environment and data within the CAE system with product changes in the subsystem through the knowledge graph. This integration facilitates reliable information interaction between the machine twin model and the product twin model, establishing a comprehensive and synchronized digital twin model for the manufacturing process. This model achieves a digital twin of the manufacturing process from multiple perspectives, enhancing transparency and efficiency throughout the manufacturing workflow. In this subsystem, users can efficiently access detailed data about specific manufacturing operations. Geometry models are neatly catalogued in a model library and integrated via the universally compatible STEP file format, ensuring seamless system integration. The geometric data from each STEP file is also managed through the knowledge graph, allowing users to easily access various parameter details with a simple click. This holistic strategy not only improves the retrieval and clarity of process information but also significantly enhances the overall efficiency and transparency of manufacturing operations.

To validate the generalizability and effectiveness of the proposed three-layer knowledge model, an experiment was conducted to compare its performance with other established methods in automatically extracting knowledge triples from unstructured data. This comparison addresses the concern regarding dependency on specific technologies and tools, ensuring that the proposed approach is versatile and adaptable across different technical environments. The experiment focuses on evaluating the ability of different methods to extract accurate and relevant triples from a dataset derived from manufacturing process documents and reports related to aero-engine blade production. The metrics used for evaluation are Precision (%), Recall (%), and F1 Score, which are standard measures in information extraction tasks. The experiment selected three comparative models: a Traditional NLP-Based Method using Open Information Extraction (OpenIE), a Deep Learning-Based Method employing a Bi-LSTM neural network, and a Hybrid Method represented by the BERT-Bi-LSTM-CRF model.

As shown in Table 2, the Proposed Three-Layer Knowledge Model outperforms the other methods across all evaluation metrics. With a Precision of 77.38%, it shows a superior ability to extract correct triples from the data. Its Recall of 83.14% indicates effectiveness in retrieving relevant triples present in the dataset. The F1 Score of 80.15% balances both Precision and Recall, showcasing overall excellence. The Hybrid Method (BERT-Bi-LSTM-CRF) achieved a Precision of 71.43%, Recall of 73.17%, and F1 Score of 72.29%, performing better than the Deep Learning and Traditional NLP methods but not surpassing the proposed model. The Deep Learning-Based Method (Bi-LSTM) recorded a Precision of 67.39%, Recall of 69.42%, and F1 Score of 68.39%, indicating moderate performance. The Traditional NLP-Based Method (OpenIE) had the lowest scores, with a Precision of 63.91%, Recall of 64.98%, and F1 Score of 64.44%, highlighting limitations in handling complex, domain-specific language without advanced modeling techniques. These results suggest that the integration of domain knowledge and cognitive reasoning in the proposed three-layer knowledge model significantly enhances its performance in knowledge extraction tasks. The model’s ability to capture complex relationships and nuances inherent in manufacturing documents contributes to its superior accuracy and generalizability. This experiment validates the effectiveness of the proposed model and addresses concerns about dependency on specific technologies, demonstrating its adaptability across different technical settings.

**Table 2:** Identification and evaluation results.

Model	Precision (%)	Recall (%)	F1 Score
BERT-Bi-LSTM-CRF	71.43	73.17	72.29
Bi-LSTM	67.39	69.42	68.39
OpenIE	63.91	64.98	64.44
Proposed Model	**77.38**	**83.14**	**80.15**

**Fig. 9:**
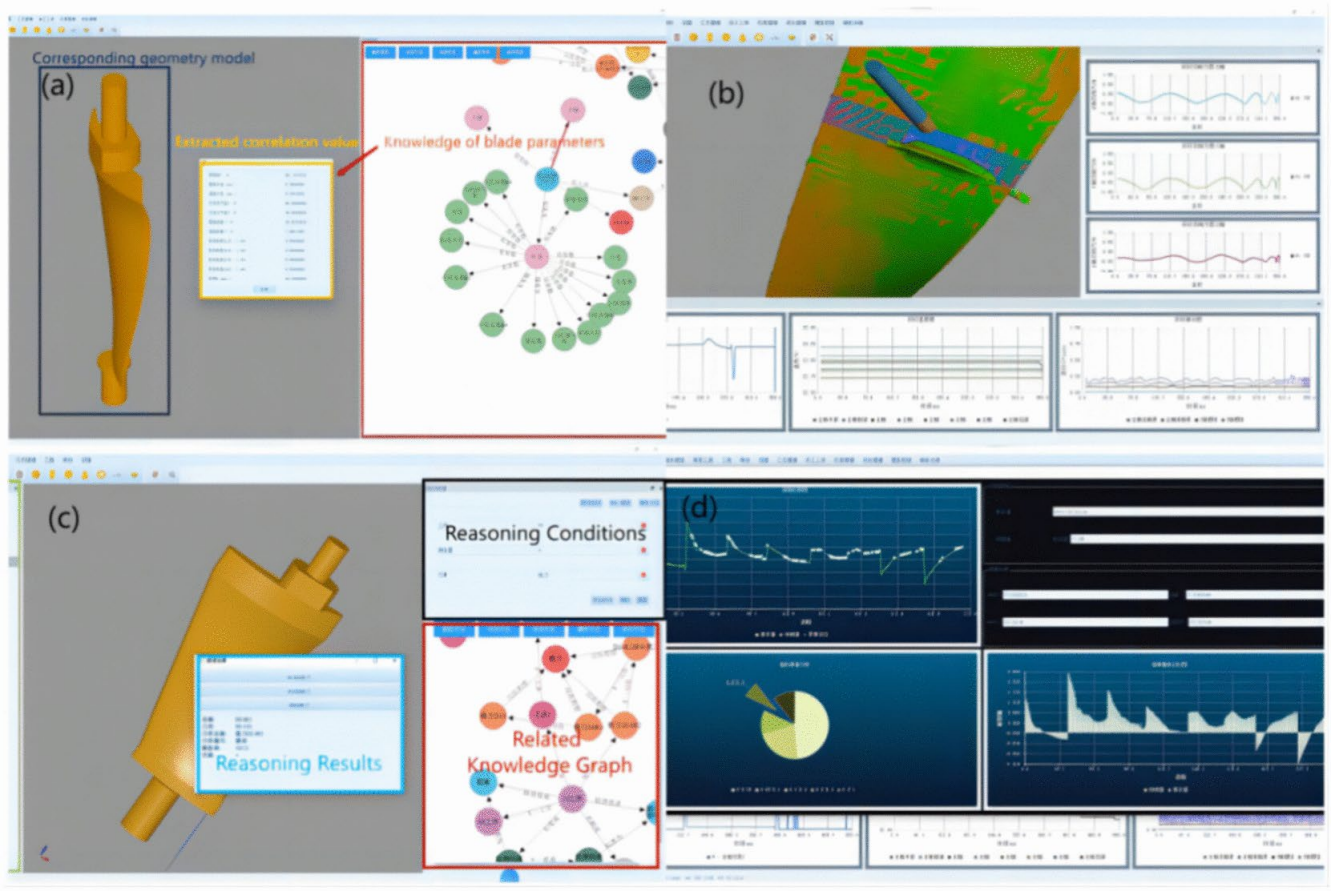
System for decision support: (**a**) Geometric model design; (**b**) Process monitoring; (**c**) Knowledge recommendation; (**d**) Prediction and analysis.

As for decision system, Fig. 9 displays the system’s various functionalities for decision knowledge. (a) demonstrates the rapid construction of a geometric model using information provided by the knowledge graph. It relies on relevant geometry classes in the model knowledge graph as its driving force. Essentially, through the knowledge graph, an interface between the model and the system’s functional modules is realized. By utilizing the element information of the model, the call for geometric modeling functions is driven, indirectly mapping the manufacturing process by expressing the product’s state. (b) showcases process simulation achieved by combining process information with dynamic simulation. By integrating process information with real-time data, a real-time data interface with the system’s relevant functions is established, simultaneously driving the call of the simulation module, achieving visualization of the manufacturing process. (c) represents process knowledge Q&A, where the image illustrates the recommendation of suitable machining tools for the process based on specific prerequisites. Its essence lies in utilizing information queries and knowledge inference from the knowledge graph to extract relevant entity relationships and attributes. By using the knowledge graph embedding model, knowledge graph completion and prediction are realized, ultimately outputting results for answering relevant questions. (d) pertains to prediction and analysis for the next manufacturing stage, presenting potential outcomes for the next phase to users. This function and knowledge inference are essentially the same. The difference lies in the fact that the training model for the prediction and analysis function will provide different information requirements to the knowledge model based on the objectives of the training model, leading to many different query results. Using these results for prediction and analysis, the final outcomes are then output.

**Fig. 10:**
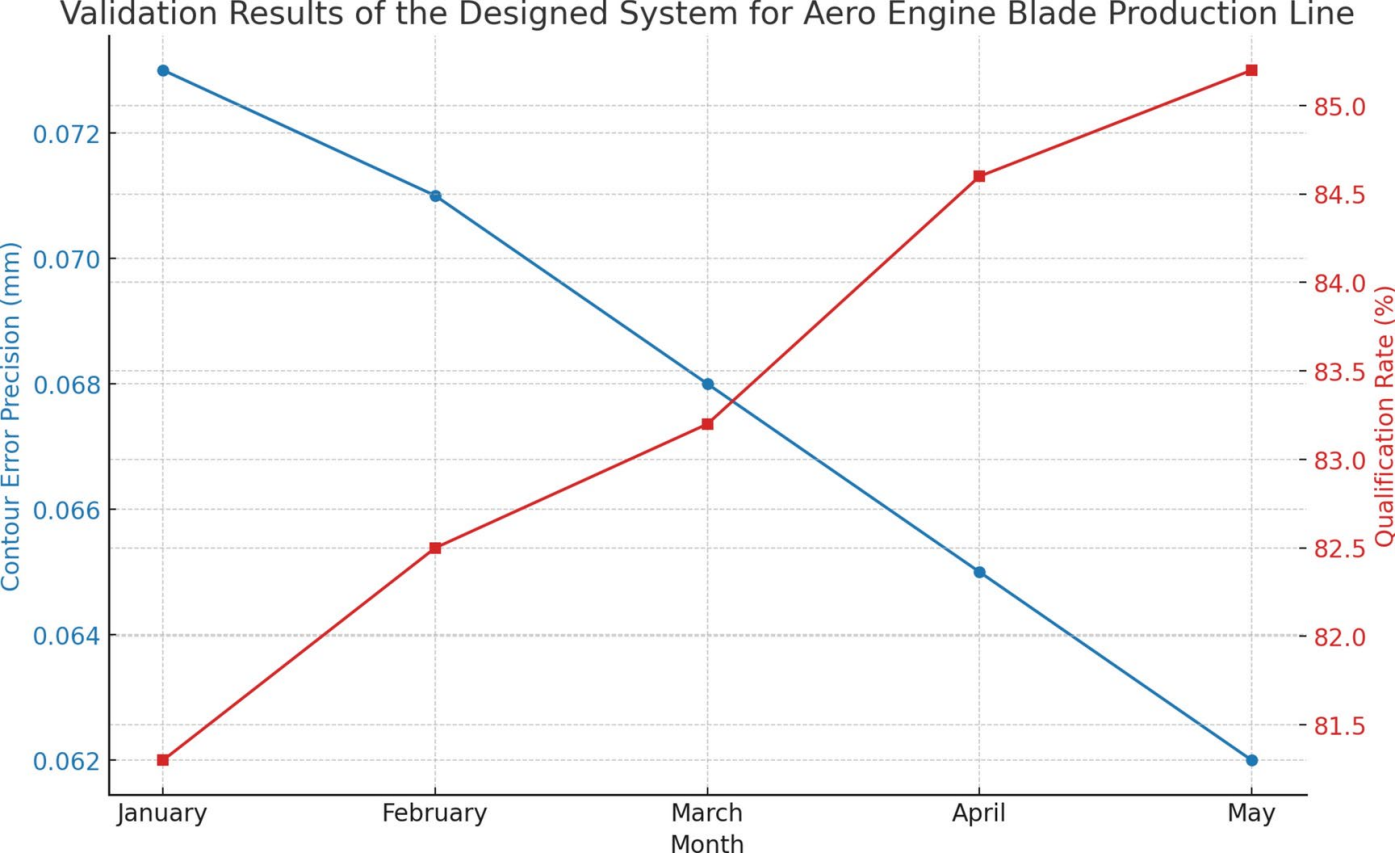
Validation results of the system for aero engine blade production line.

In an enterprise’s aero engine blade production line, our designed system was validated over a period of 5 months (2024), covering a total of 200 batches of blade products. Figure 10 shows the validation results, including two key indicators: the maximum contour error precision of the blades and the product qualification rate. During the validation period, the maximum contour error precision of the blades gradually decreased from 0.073mm in January to 0.062mm in May. The specific error precisions for each month are as follows: January was 0.073mm, February was 0.071mm, March was 0.068mm, April was 0.065mm, and May was 0.062mm. This result indicates that our designed system significantly improved the processing precision of the blade contours during production, reducing the error further. At the same time, the product qualification rate increased from 81.3% in January to 85.2% in May. The specific qualification rates for each month are as follows: January was 81.3%, February was 82.5%, March was 83.2%, April was 84.6%, and May was 85.2%. This demonstrates that our system not only performed excellently in improving processing precision but also significantly enhanced the overall quality of the products. The significant improvements in these two key indicators show that the application of our designed system in the aero engine blade production line was very successful, greatly increasing production efficiency and product quality. This further proves the effectiveness and reliability of our system, providing strong technical support for enterprises in high-precision production and quality control.

**Fig. 11:**
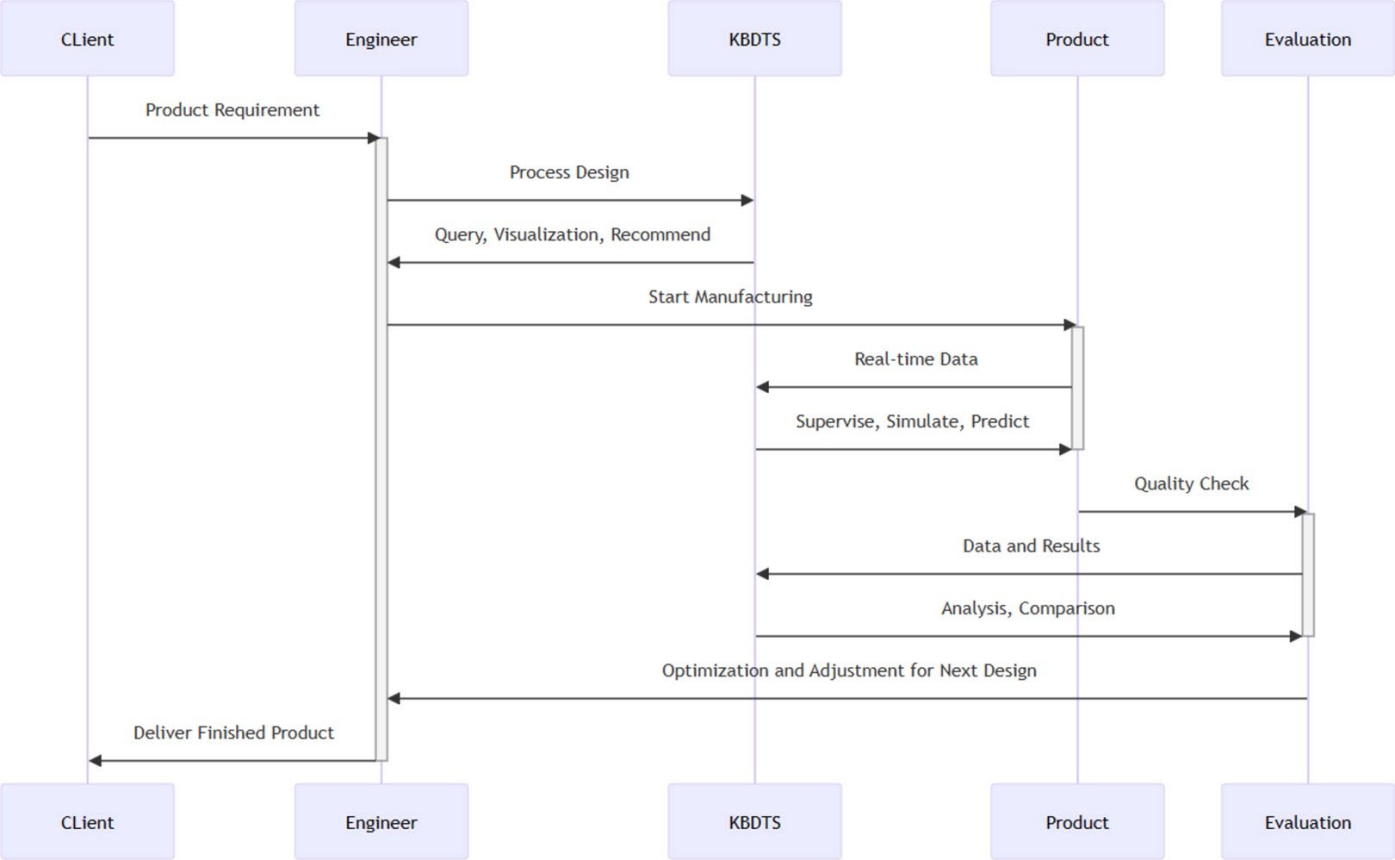
Three layer knowledge graph covers the entire manufacturing process.

As depicted in Fig. 11, Throughout the entire lifecycle of the manufacturing process for aero- engine blades, three layer knowledge graph consistently offers its services. Upon receiving the client’s requirements, engineers utilize three layer knowledge graph to assist in the process design phase. Trough its querying, visualization, and recommendation features, aids engineers in designing the geometric model of the blade. It also provides more rational and scientific suggestions for detailed design and blueprint drafting, and offers guidance in the selection of raw materials. During the manufacturing phase, by capturing real-time data generated in the processing, three layer knowledge graph offers supervision, simulation, and prediction functions, ensuring the smooth progression of the production process. In the evaluation phase, it analyzes and compares the results and data from product inspections, assisting engineers in discussions and in drawing conclusions from their experiences, adjusting and optimizing the design for the next cycle. This iterative process guarantees continuous improvements in product quality and production efficiency. Ultimately, the blade that meets customer requirements is successfully delivered.

## Discussion

This study, through the practical example of manufacturing aero-engine blades, demonstrates the application of a three-layer architecture knowledge graph in the modern manufacturing industry, particularly in the construction of digital twin models. This three-layer architecture consists of the concept layer, model layer, and functional layer, each designed to meet different needs within the manufacturing process and collectively supporting the digital twin modeling of complex products. The successful application of this system in the aero-engine blade production line suggests broader implications for its use in other complex manufacturing environments. By leveraging the knowledge graph’s structured and interconnected information, manufacturers can achieve higher precision and quality in their production lines.

These results demonstrate the effectiveness of the three-layer architecture knowledge graph in addressing practical challenges in manufacturing. The concept layer provides a foundational understanding and representation of manufacturing knowledge. The model layer translates this knowledge into actionable models that guide the manufacturing processes. Finally, the functional layer implements these models into the digital twin system, enabling real-time monitoring, simulation, and optimization of the manufacturing process. However, several challenges and limitations were encountered during implementation, highlighting areas for future research and improvement:

- Data Integration Difficulty: Effectively integrating data from various sources and formats into the knowledge graph remains a significant challenge. The heterogeneity of data—including differences in data structures, formats, and semantics—can lead to information loss or errors, impacting the accuracy and reliability of the model. Future work could explore advanced data integration techniques, such as developing universal data exchange standards, employing semantic data modeling, and utilizing ontology alignment methods to enhance compatibility and reduce integration complexity.
- Real-Time Data Processing Capabilities: Although the functional layer is designed to handle real-time data, efficiently processing and utilizing large volumes of real-time data continues to be a technical challenge. High throughput and low-latency requirements necessitate robust computational resources and optimized algorithms. Future research could focus on developing scalable real-time data processing frameworks, leveraging edge computing, parallel processing, and advanced data compression techniques to improve performance and efficiency.
- System Scalability and Adaptability: As manufacturing processes evolve and new technologies are introduced, the digital twin system requires continuous updates and maintenance to adapt to new production environments and demands. Ensuring scalability without compromising system performance is critical. Future developments could include modular system designs and adaptive algorithms that allow the digital twin to scale seamlessly and adjust to changes in the manufacturing landscape, including incorporating new equipment, processes, and materials.
- Dependence on Expert Knowledge: While the knowledge graph utilizes the expertise of domain experts, over-reliance on expert input may limit the system’s universality and flexibility. This dependence can also slow down the system’s ability to adapt to new information or technologies. Future efforts could focus on integrating machine learning and artificial intelligence techniques for automated knowledge extraction, learning from operational data, and employing natural language processing to reduce reliance on manual expert input, thus enhancing the system’s ability to generalize across different contexts.

Addressing these limitations is essential for realizing the full potential of the three-layer cognitive digital twin model in smart manufacturing. By focusing on these areas, future research can further optimize the digital twin system, leading to broader applicability across various industrial contexts. Overcoming these challenges will contribute to the advancement of intelligent manufacturing, enabling more efficient, flexible, and responsive production systems that align with the goals of intelligent manufacturing.

## Conclusion

This research successfully demonstrates the utility and benefits of a three-layer architecture knowledge graph in constructing digital twin models, particularly validated through a specific case study involving the manufacturing of aero-engine blades. By integrating the concept layer, model layer, and functional layer, the study not only accurately maps the manufacturing process but also achieves dynamic optimization and real-time monitoring, enhancing production efficiency and product quality.

The concept layer provides a robust ontology library and rule constraints, formulated from the knowledge of domain experts and technical manuals, which furnish the digital twin model with a rich and precise conceptual definition and framework. This structured approach not only aids in standardizing data and processes but also ensures the overall consistency and scalability of the model. The model layer, by instantiating the rules and concepts defined in the concept layer, forms a tangible and operational digital twin model. The focus of this layer is on translating theory into practice by intricately simulating the manufacturing process of specific products, enabling the model to precisely map the characteristics of the physical world. The decision layer then performs advanced analysis and optimization tasks based on the outputs from the model layer. In this study, the implementation of the decision layer includes dynamic optimization, maintenance forecasting, and risk management, all facilitated through advanced algorithmic models aimed at enhancing production efficiency and product quality. Moreover, the digital twin system demonstrated in this study shows that as new data is continuously integrated, the model can be constantly updated and improved, thereby continually optimizing the manufacturing process. This iterative model update not only improves the adaptability of operations but also enhances the system’s responsiveness to changes.

In conclusion, the three-layer architecture knowledge graph provides a powerful methodological framework for digital twins, making operations more efficient and precise during the digital transformation process. Future research could further explore how to expand this framework to other complex manufacturing scenarios to promote the widespread application of smart manufacturing and achieve broader industrial optimization and innovation.

## Data Availability

The data that support the findings of this study are available from Aecc South Industry Company Limited, but restrictions apply to the availability of these data, which were used under license for the current study, and so are not publicly available. Data are however available from the authors upon reasonable request and with permission of Aecc South Industry Company Limited [Contacts: Mr.Han;E-mail: yonghan@ouc.edu.cn].
